# Correction: Computational investigations of click-derived 1,2,3-triazoles as keystone ligands for complexation with transition metals: a review

**DOI:** 10.1039/c8ra90107d

**Published:** 2019-01-04

**Authors:** Tayebeh Hosseinnejad, Fatemeh Ebrahimpour-Malmir, Bahareh Fattahi

**Affiliations:** Department of Chemistry, Faculty of Physics & Chemistry, Alzahra University Vanak Tehran Iran tayebeh.hosseinnejad@alzahra.ac.ir +98-21-8804-1344 +98-9124775800

## Abstract

Correction for ‘Computational investigations of click-derived 1,2,3-triazoles as keystone ligands for complexation with transition metals: a review’ by Tayebeh Hosseinnejad *et al.*, *RSC Adv*., 2018, **8**, 12232–12259.

The authors wish to publish this correction to correctly attribute work cited in their review article.

A citation to the work of Schweinfurth *et al.*, ref. 55 in the original article, should have been included at the end of the first paragraph on page 12241. The sentence “The energies of…” should be changed to “The energies of the spin-allowed d–d transitions of complexes 23′, 24′, and 26′ were calculated comparatively based on their X-ray and DFT-geometry-optimized structures^55^”.

On page 12242, the second paragraph beginning “To assess the…” should be changed to “To assess the kinetics of the isomerization procedure theoretically, Pinter *et al.* focused on the determination of the transition state (TS) connecting 27 and 28*via* a one-step mechanism,^57^ as illustrated in Fig. 1. They showed that the calculated activation barrier values and the entropy contributions were in a good agreement with the experimental data”.

The caption for Fig. 1 should be changed to “Fig. 1 One-step mechanism for the isomerization of 27 into 28. It should be noted that the figure has been redrawn using data from ref. 57”.

The Royal Society of Chemistry apologises for these errors and any consequent inconvenience to authors and readers.

## Supplementary Material

